# Immunization against Small Ruminant Lentiviruses

**DOI:** 10.3390/v5081948

**Published:** 2013-08-02

**Authors:** Ramsés Reina, Damián de Andrés, Beatriz Amorena

**Affiliations:** Institute of Agrobiotechnology, CSIC-UPNA-Government of Navarra, Ctra. Mutilva Baja s/n, Navarra 31192, Spain; E-Mails: ancad@unavarra.es (D.A.); bamorena@unavarra.es (B.A.)

**Keywords:** small ruminant lentivirus, Maedi-Visna, immunization

## Abstract

Multisystemic disease caused by Small Ruminant Lentiviruses (SRLV) in sheep and goats leads to production losses, to the detriment of animal health and welfare. This, together with the lack of treatments, has triggered interest in exploring different strategies of immunization to control the widely spread SRLV infection and, also, to provide a useful model for HIV vaccines. These strategies involve inactivated whole virus, subunit vaccines, DNA encoding viral proteins in the presence or absence of plasmids encoding immunological adjuvants and naturally or artificially attenuated viruses. In this review, we revisit, comprehensively, the immunization strategies against SRLV and analyze this double edged tool individually, as it may contribute to either controlling or enhancing virus replication and/or disease.

## 1. Introduction

Small Ruminant Lentiviruses (SRLV) cause by far the most prevalent lentiviral infection in the world, affecting sheep and goats from Europe, America, Africa, Asia and Australia [1]. SRLV include classical Visna/Maedi (VMV) and Caprine Arthritis Encephalitis (CAEV) viruses, a group of viruses showing one of the highest heterogeneity in terms of genetics, host range, immune activation and disease outcome. VMV and CAEV were originally believed to be specific to the sheep and goat species, respectively, but currently, several decades after their discovery, they are being considered as a single major group of viruses, SRLV, following HIV phylogenetic classification standards [2]. So far, five genotypes have been described within SRLV, genotypes A and B, corresponding to VMV and CAEV prototypes, respectively, and genotypes C, D and E [2,3]. SRLV are closely related, not only from the genetic point of view. The natural transmission of SRLV between sheep and goats constitutes a unique feature among lentiviruses that points out the need to reconsider diagnostic, as well as vaccination strategies jointly, in order to gain sensitivity and protection against heterologous strains in both species [4]. 

Animals are mainly infected either by ingestion of infected colostrum/milk or by direct contact with infected animals through respiratory secretions [5]. After cell infection, main targets (monocyte/ macrophage lineage) present viral antigens to the immune system, and antibodies may become readily detectable in serum one month after infection. These antibodies and other immune system effectors control viraemia transiently, until viral replication rebounds, associated with macrophage maturation and production of pro-inflammatory and pro-attractive cytokines that recruit T-cells to the replication site. This mechanism leads to the typical interstitial T-cell infiltration and follicular hyperplasia present in SRLV-associated lesions. In spite of the strong immune response elicited to control viral replication, the virus continues its spread. Infected macrophages trigger infiltrative and inflammatory processes that lead to multi-organ dysfunction. Thus, lesions present in affected tissues are considered to be immunomediated [5]. Accordingly, immune responses against SRLV and, also, other lentiviruses constitute a double edge sword, since they may contribute to viral containment in long-term non-progressors or, rather, to lesion development in most of the animals. Immune correlates useful for distinguishing an effective immune response from a detrimental one need to be fully elucidated. 

Icelandic researchers discovered the first lentivirus, VMV, and established the concept of “slow infections” and the VMV as the prototype agent for lentiviral infections, until the appearance of HIV [6,7]. Following discovery, VMV and, the whole SRLV group, together with simian immunodeficiency virus (SIV), feline immunodeficiency virus (FIV) and equine infectious anemia virus (EIAV), became useful for developing practical animal models for vaccination trials against lentiviral infections. Classically, FIV and HIV infect CD4+ T cells, causing their depletion and an acquired immunodeficiency syndrome (AIDS), whereas individuals infected by EIAV and SRLV (which do not infect T-cells) have been considered for years non-immunodeficient. However, exhaustive research on HIV and FIV infections has led to the description of a mechanism inducing AIDS unrelated to CD4+ T-cell counts and involving anergy [8]. In line with this, SRLV infection leads to IgG1 antibody response [9], Th1 impairment [10] and diminished B7 molecule expression, likely contributing to the detected decreased proliferative responses and reduced delayed-type hypersensitivity (DTH) reactions [11,12]. This type of immunodeficiency could explain the elevated susceptibility of SRLV infected animals to other secondary infections. 

Despite optimal research on immunology and vaccinology against lentiviral infections, no satisfactory treatment or vaccine inducing sterilizing immunity has been developed so far. Immunization trials against SRLV have included highly innovative and promising strategies, from live vaccines to DNA plasmid delivery using a gene gun, going through the infection with attenuated live vaccines.

Deviating immune response towards the Th1 profile appears to be essential for reaching a protection strong enough to ensure, if not sterilizing immunity, at least partial protection against lentiviral infections [5]. Although neutralizing antibodies have been proposed as crucial in protection against HIV or SIV, studies on SRLV infection strongly suggest that SRLV-specific neutralizing antibodies are not protective. Rather, they could provide a marker for a Th2 non-protective response or, even, favor infection after exposure to experimental infection. Several issues are still to be solved in SRLV immunization, including viral heterogeneity, immune modulation by viral infection, detrimental immune responses or induction of long-lasting immunity, among others.

In this review, we aim to summarize the most relevant studies regarding SRLV immunization in the last few years, which have provided promising results in terms of effective immune activation and/or partial protection upon viral challenge. 

## 2. Protein Vaccines

The vaccination history against SRLV begins with the first attempts using whole virus inactivated by different methods, such as heat, formalin or polyethyleneimine, and employed without adjuvant, with Freund incomplete adjuvant or with aluminum hydroxide to vaccinate sheep. These formulations induced the production of precipitating antibody against the virus, but did not correlate with protection against challenge. Instead, the high antibody production worsened lentivirus-induced arthritis [13].

Similar immunization experiments were performed using purified CAEV obtained from tissue culture, inactivated with formalin and formulated with Freund’s complete adjuvant, which resulted in more severe arthritis that developed more rapidly [14]. The therapeutic potentialities of this formulation were also tested following intraarticular inoculation of inactivated CAEV into persistently infected goats that had developed the greatest carpal joint swelling. In spite of the huge antibody production in immunized animals, viral challenge exacerbated lentivirus-induced arthritis.

Following these experiments, SRLV recombinant proteins became available and were tested in adjuvant formulations. The objective was to provide viral protein to antigen presenting cells, which would present processed epitopes to CD4 T cells in the major histocompatibility (MHC) class II context, inducing antibody responses. Specifically, envelope protein from strain CAEV-63 was administered intramuscularly using immunostimulating complexes (ISCOM) and Quil A Saponin. Neutralizing antibody production was then evaluated *in vitro* against heterologous CAEV strains using sera from immunized animals. As expected, antibody production was high, and neutralizing antibodies against the heterologous strain were detected; but, protection against *in vivo* challenge was not assessed. In spite of the production of neutralizing antibodies, some animals centered the response against other non-neutralizing immunodominant epitopes, likely enhancing viral infection [15]. This was the first attempt to potentiate protection against heterologous challenge, which is a key point when designing a vaccine against the highly heterogeneous SRLV group.

There is strong evidence that virus-specific CD4 T-cell responses are crucial to control persistent infections caused by lentiviruses [5]. In order to better stimulate this cell subpopulation, Freund’s incomplete adjuvant (FIA) mixed with a synthetic peptide from CAEV-GAG protein encompassing a CD4 T-cell epitope was used to immunize three caprine leucocyte antigen (CLA)-haplotype defined groups of goats. T-cell proliferative responses were stronger in vaccinated animals, although they had a transiently higher proviral load compared to controls at early stages. No relationship was found between CLA and the ability to control virus replication or between viral load and production of IFN-γ or IL-4, but a clear correlation between viral load and expression of granulocyte-monocyte colony stimulating factor (GM-CSF) was observed in the early phase of infection, when macrophage maturation occurs together with viral replication [16].

Overall, these studies point out that great care should be taken when designing vaccine candidates against lentiviruses. Indeed, SRLV might have developed a yet unknown strategy to manipulate the immunological response of CD4^+^ T-cells, either directly or by affecting the expression of immunomodulators in infected antigen-presenting cells, as already shown for macrophages [17,18]. Depending on these modulators, the expression of specific molecules on the surface of macrophages could affect permissiveness to certain strains, as suggested in recent studies [19]. 

## 3. Live Attenuated Vaccines

After the first discouraging trials with inactivated virus, live attenuated vaccines clearly stimulated vaccine research against SRLV in the 1990s. Attenuated virus vaccines have been assessed for the prevention of a wide range of viruses, such as influenza virus [20], SIV [21], EIAV [22], chicken pox or yellow fever viruses [23], leading to different degrees of protection. FIV vaccines based on modified attenuated live viruses induce moderate levels of cellular immunity and significant antibody responses, conferring increased protection rates, compared with subunit vaccines [24]. Regarding EIAV, multiple immunization strategies have been explored, but among attenuated viruses, inactivated virus particles, protein subunits, DNA vaccines and live vectors, the highest level of protection has been reached when using attenuated viruses, likely due to the continuous antigen exposure and optimized maturation of the immune response. However, there has always been an inverse relationship between the level of protection and the level of attenuation, indicating that a minimal replication rate is needed for eliciting a protective immune response in these strategies. In line with this, an EIAV attenuated strain (DLV120) obtained by *in vitro* passages in donkey cells, which confers protection against EIAV challenge [25,26,27,28], has been extensively used in China with promising results [22]. However, antigenic variation poses a major obstacle to lentivirus vaccine development, since ENV-based vaccines are highly effective, and there is an inverse correlation between the level of protection and the level of sequence variation (high in ENV sequences) between the challenge and the vaccine strains [29,30]. Thus, previous knowledge on circulating strains would be essential for designing an effective vaccine against EIAV. 

The use of SRLV attenuated viruses obtained by deletion of selected genes, mainly *vif*, *tat* and *dUTPase*, also looks promising. The protective role these mutants play has been determined using conventional or DNA vaccination, which may prevent variation in infection efficacy and allows genetic manipulation to generate partially deleted retroviral genomes. In a series of studies, Harmache and his group demonstrated that *vif*- and *tat*-deleted genomes could be used as live attenuated vaccines [31]. However the corresponding deletions affected the viral replication in different forms. The *vif-* deleted viruses were highly attenuated [32], whereas the *tat*-deleted ones were slightly attenuated [33]. Immunization with *vif*-deleted viruses failed to protect goats against homologous pathogenic challenge, since arthritis was evident in spite of a significant antibody production [31]. SRLV *tat* gene was found dispensable for viral replication, since persistent infection was achieved upon transfection with a *tat*-deleted CAEV Cork molecular clone in caprine cells. Furthermore, the induced seroconversion after *in vivo* direct inoculation of the *tat-*deleted proviral DNA, as well as the isolation of the virus, further confirmed persistent infection [31]. Priming with the *tat*-deleted CAEV Cork molecular clone resulted in partial protection against CAEV Cork infection, as the challenge virus was undetectable in protected animals. However, this vaccine was still pathogenic, since histopathological changes were observed in joints from CAEV *tat*-immunized animals, suggesting the avoidance of its use in the field [34]. However, it is not fully clear whether these mild lesions are reflecting virus-induced damage or, rather, if they may represent a curative transient inflammation towards recovery. 

Icelandic researchers explored, subsequently, the use of a low pathogenic molecular clone, which differed only by 1% in nucleotide sequence from a highly pathogenic molecular clone, both obtained from Icelandic infected sheep using molecular biology engineering [35]. Intratracheal inoculation with high doses (10^7^ TCID_50_) of the low pathogenic clone induced low antibody titers, suggesting a Th1-biased response. Although the vaccine failed to protect sheep against intratracheal challenge with 10^3^ TCID_50_ of the highly pathogenic molecular clone, decreased numbers of viral isolations and milder lesions were observed in immunized animals, suggesting a diminished viral load compared to controls [36]. Furthermore, these experiments clearly confirmed the inverse correlation between the level of attenuation and the degree of protection. 

Compared to wild-type viruses, dUTPase-deficient viruses have also shown an attenuated profile with similar delays between infection and seroconversion, similar frequencies of virus recovery from both blood-derived and mammary-derived macrophages, similar rates of divergence and similar organ distributions of viral RNAs, but milder severity in the mononuclear cell infiltrations of the synovia [37]. However, no vaccination studies have been conducted with these artificially deleted mutants to explore if the level of attenuation is adequate.

This information encouraged the assessment of genotype E of SRLV with natural deletions in the dUTPase and *tat* genetic regions, as a candidate to protect against SRLV infections. Genotype E1 was firstly discovered in the Roccaverano goat breed from Piedmont (Italy) as a highly divergent SRLV [38]. Analysis *in vivo* showed that although the virus does not cause detectable pathological signs; genotype E is prevalent and has been hiding from standard serological tests, due to its highly divergent structural proteins, and has also been inadvertently undetected by breeders and practitioners, due to the absence of symptoms. However, genotype E-specific serological tests have revealed 45% seropositivity in goats from the Sardinian island [39]. Interestingly, genotype E1 (non-pathogenic) and B (highly pathogenic) co-infected goats do not show any clinical sign, suggesting a possible genotype E1-mediated natural protection against pathogenic strains. Interestingly, until these findings were published, the Roccaverano breed was believed resistant to CAEV infection, as proposed in other areas of the world with other indigenous breeds [38,40].

To determine if Roccaverano infection could protect from heterologous infection by immune mechanisms, goats were infected with Roccaverano (E1) and challenged with CAEV Cork (genotype B) strains. Animals infected with strain Roccaverano developed strong antibody and proliferative T-cell responses exclusively mounted against homologous antigens, arguing for a non-immune protective mechanism. However, the cytotoxic T lymphocyte (CTL) response of E1 infected animals was exclusively directed against heterologous-strain (B) infected cells and not against homologous-strain (E1) infected cells [41]. This feature could explain both, the level of protection achieved and the lack of infiltrates in Roccaverano infected goats. Furthermore, infection with Roccaverano strain protected goats from CAEV Cork challenge in terms of proviral load, lesion development and transmission to the offspring [42]. Although this may constitute a step forward in SRLV control, no sterilizing immunity was achieved, since CAEV Cork provirus was detectable in immunized animals. There are also some issues to be elucidated, for example, whether Roccaverano infection could protect against other genotype B strains or, even, against other genotypes.

## 4. Plasmid and Vaccinia Immunization Strategies

Plasmid DNA immunization induces both strong humoral and cellular immune responses against multiple infections in different hosts. Following DNA uptake, cells will transcribe and produce the encoded proteins. In this way, antigens are presented, mimicking natural mechanisms. The inoculated plasmids may also encode any interesting molecule that enhances or modulates the immune response. The efficiency of DNA uptake is critical to reach acceptable antigen levels for triggering an immune response. In this regard, different methods have been developed to improve transfection efficiency, based on the administration of DNA in the context of liposomes, cationic lipids, nanoparticles or gold beads. 

Cheevers and collaborators [43] obtained promising results by applying intradermal genetic immunization against SRLV using *env*- and *tat*-naked plasmids together with immunomodulatory plasmids encoding caprine IFN-γ. Firstly, they found that goats administered with pUC-CAEV*env* plasmids mounted Th1 responses, validating this strategy against SRLV infection. In addition, they characterized immune responses to CAEV ENV antigen in terms of quality and quantity of antibody production or cytokines involved and, also, evaluated the possibility to redirect immune response by using immunomodulatory components, such as IFN-γ encoding plasmids [43]. Goats primed with *env* plasmids and boosted with purified ENV in FIA did not develop severe arthritis after challenge with pathogenic CAEV Cork, whereas control groups showed evident lesions in carpal joints 428 days after boosting. In addition, antibody response was suppressed, and proviral load in prescapular lymph node, as well as virus recovery rates were decreased in vaccinated animals, suggesting suppression of viral replication [44]. However, sterilizing immunity was not achieved, since vaccinated animals were not protected from infection, even though they were able to mount a defense against the challenge virus, a common finding in prime-boost vaccine trials. 

Prompted by these quite encouraging results, different European research groups, including ours, began to explore the possibility of immunizing against VMV through gene delivery. Firstly, *env* from VMV strain Ev1 was administered in the presence of IFN-γ encoding plasmids to sheep in the vaginal mucosa using a gene gun. Specifically, DNA plasmids precipitated onto gold particles, individually or mixed, were bombarded through the vaginal mucosa. This strategy was undertaken to improve DNA uptake, since Langerhans cells of the sub-epithelium are supposed to be directly transfected upon gene gun delivery [45]. Indeed, the amount of plasmid needed is significantly lower compared to naked DNA strategies. Following immunization, sheep developed a protective immune response in terms of isolation of the virus, proviral load in blood and the presence of challenge virus assessed by PCR. However, this protection against strain Ev1 was not long lasting, since two years post-challenge, the presence of challenge virus in immunized animals was evidenced by PCR/sequencing. Interestingly, this protection was inversely related to the production of neutralizing antibodies in the animals and was not related to the MHC antigens assessed (OLA DRB-I). Although association has been found between CLA-antigen and SRLV-arthritis susceptibility in Saanen goats [46,47], so far, no association between host genetics, MHC or CLA genotyping, and the capacity to restrict SRLV infection has been reported in immunization studies [16,45]. However, employing new generation sequencing, TMEM154, an ovine transmembrane protein, has been associated to reduced/increased susceptibility to SRLV infection, even though resistance was not complete [48,49]. In this context, a CCR5 deletion located in an intron that modifies the levels of protein expression has been related to reduced susceptibility to infection [50].

The *env* gene has demonstrated, so far, the ability to immunize sheep and goats and to confer partial protection against challenge with homologous virus. However, due to the hypervariability of this region, protection against relatively distant genotypes is still debatable. In addition, while involvement of antibodies in protection against other lentiviral infections, such as HIV, appears to be widely accepted, in SRLV infection, evidence for the contrary is accumulating. Therefore, a series of studies have been performed to test the efficacy of the *gag* gene (more conserved among strains) in search of immune responses correlating with protection, likely Th1. Together with plasmid inoculation, recombinant vaccinia virus or adenoviruses have been incorporated in prime/boost immunization strategies that would help to develop a strong antibody response. 

In sheep, mucosal priming using plasmids conjugated with polyethylenimine particles (PEI) and boosting with modified vaccinia Ankara virus expressing SRLV *gag* and/or *env* resulted in the development of faint humoral and cellular responses against the challenge strain [51]. Immunity was not sterilizing, and disease was not clearly prevented. Protection was achieved in terms of decreased proviral load in *env*-immunized animals. However, like in other studies describing protection, pathology was not reduced in the *env*-immunized animals. On the other hand, *gag*-immunized animals showed a reduced pathology score, likely reflecting the limited immune response elicited with this strategy [51].

Alternatively, when *env* and *gag* genes were delivered by epidermal bombardment using the same prime/boost strategy [52], cellular and humoral immune responses became stronger compared to that obtained with PEI administration and comparable to those obtained after gene gun delivery in vaginal mucosa [45]. Particularly, postchallenge CTL responses were significantly increased in *env*- and *gag-env*-immunized groups. Challenge virus was detectable in almost all immunized animals, but *gag* and *gag-env* groups showed a decreased proviral load in blood and mediastinal lymph node, respectively. In terms of pathology, *env* groups showed an increased pathology score, and other groups did not differ from the control group. The pathology score observed after immunization could reflect the immune response activation or, rather, the pro-inflammatory processes occurring along viral infection. Which of these processes took place could not be elucidated in these experiments, due to the short period from challenge to slaughtering (at 84 days post-challenge) [52] compared to other plasmid vaccination studies [44]. 

B7 costimulatory molecules expressed on the surface of antigen-presenting cells provide the essential second signal in the immunological synapsis for effective antigen presentation and proper T-cell clonal expansion [53,54]. Previous to their use as adjuvants in vaccination studies [12], we had shown that ovine B7 molecules are upregulated in infected asymptomatic animals, in contrast with clinically infected animals. These molecules may, thus, improve effective antigen presentation and prevent anergy development, which encouraged their use in the immunization inoculum. Accordingly, the most promising results within this series of prime/boost immunizations were obtained with the inclusion of plasmids encoding costimulatory molecules besides *gag*-*env* genes, all delivered by gene gun bombardment. Immune responses were similar in potency and quality in *gag* plus *env*-immunized groups, but inclusion of both B7 molecules (CD80 and CD86) empowered antibody production and anticipated CTL responses. Most importantly, the challenge virus was only detectable by sensitive PCR in half of the immunized animals that received both B7 molecules, it being the first study showing infection clearance [55]. However, as highlighted before in plasmid immunization and, also, in vaccination studies with attenuated viruses, the pathology score was, again, increased in the most reactive groups, even those showing no detectable virus. This further supports the idea that SRLV-related lesions after stimulating the immune system should be carefully interpreted. Infiltration and inflammation detected in target tissues of immunized animals might reflect the immune response elicited by these animals, rather than tissue damage derived from viral infection. Unfortunately, whether these inflammatory signs would have eventually disappeared remains unknown, since pathology evaluation was, again, performed only 12 weeks after challenge.

One of the possible problems of the immunization protocols discussed so far may have been the limited number of booster immunizations involved. With this in mind, a strategy based on a combination of plasmid and protein delivery using the *gag* gene alone repeatedly during 2.5 years followed by a booster immunization with GAG precursor was implemented, inducing antibody and T-cell proliferative responses that failed to protect against challenge with pathogenic VMV. Strikingly, the virus was more easily isolated from vaccinated sheep compared to control animals, suggesting that immune responses induced by this vaccination strategy enhanced viral replication [56].

Besides the natural host, mice immunization models have also been investigated. The main limitation is murine resistance to SRLV infection, so that protection cannot be measured. Genetic immunization with plasmids was first described to direct the immune response towards a Th1 profile in mice, but extrapolation to larger animals may not always mirror this profile. Mice have been immunized with optimized plasmids for expression of p16, p25 or the entire GAG protein through injection of naked DNA. The vaccine candidate, based on p16 and p25, was able to enhance humoral responses, which, as shown by several groups, do not correlate with immune protection. Instead, immunization with the entire GAG protein did not result in production of antibodies, but unfortunately, cellular responses were not measured. Interestingly, elimination of inhibitory nucleotide sequences (INS) in the p16 nucleotide sequence slightly reduced protein expression *in vitro*, but induced higher antibody responses *in vivo* [57]. 

Overall, plasmid DNA immunization alone or in combination using prime/boost strategies induced competent Th1-biased immune responses together with the production of antibodies and conferred an ability to control homologous challenge in terms of viral load, but often without achieving sterilizing immunity. So far, antibody response has provided more disadvantages than benefits in controlling SRLV infection. Nevertheless, protection from inflammatory responses has not been achieved, and most short-term studies have been unable to discriminate between an effective immune response and virus-induced inflammation. In spite of the fruitful contributions yielded against homologous challenge, plasmid immunizations may still present some limitations, such as protection against heterologous challenge, an important feature when dealing with viruses subjected to high antigenic variation, even able to cross species barriers, such as SRLV.

Vaccinia recombinant viruses have also been used alone or in combination with other strategies as mentioned above. This model is especially recommended when antibody responses are needed and is currently being used in vaccination trials against HIV [58]. The only study in which recombinant vaccinia expressing *env* from CAEV was assessed demonstrates Th-2 biased responses with high antibody production, even when IL-12 was included in the inoculum, but failed to protect against challenge, since lesions were as evident in vaccinated as in control animals [59]. 

Comparatively, plasmid DNA administration induced a Th1-biased response with the production of IgG2, suggesting that biasing to Th1 responses highly depends on the delivery type, rather than by providing Th1-immunomodulatory cytokines [60].

## 5. Pseudoviruses

Another immunization approach is based on pseudotyped vectors, which result in the production of pseudovirions expressing the desired viral proteins, but being unable to generate progeny pseudovirions. Hence, this strategy uses only one cycle of infection, thus avoiding persistent infections. Pseudovirions contain normal GAG and POL structural proteins and may present a pantropic ENV, such as the vesicular stomatitis virus protein G (VSV-G) or a lentiviral ENV protein. In pseudovirions, the lentiviral genome has been either substituted by a marker gene or deleted to inactivate progeny formation. In this strategy, the viral antigen is exposed, and the immune response takes place, but is not exacerbated. In addition, the presentation pathways following the administration of the pseudoviruses mimic those conducted with natural live viruses, since directly transduced cells will process antigen endogenously, and single particles can be phagocytized and processed as an exogenous antigen, stimulating both cellular and humoral responses.

Using this methodology, a lentiviral DNA vaccine against HIV driven by LTR of CAEV has been applied, reaching high expression levels and inducing high IFN-γ responses in mice and macaques. Pseudovirions controlled by CAEV LTR do not require TAT transactivation and are not functional for integration mediated by HIV integrase, increasing vaccine safety and efficacy [61].

Pseudotyping other retroviruses, such as murine leukemia virus (MLV), with ENV from SRLV [62] has been as frustrating as using VMV cores pseudotyped with VSV-G [63]. However SRLV pseudotyped vectors have become available and could be explored [19,64,65] in future vaccination studies. 

## 6. Alternative Immunity to Control SRLV

Innate immune response is gaining interest in the defense against lentiviral infections. Many research groups have centered their attention on restriction factors of the intrinsic immunity, able to directly recognize viral motifs and lead to their degradation. The most studied are the tripartite motif 5 (TRIM5) and apolipoprotein B mRNA-editing, enzyme-catalytic, polypeptide-like 3 (APOBEC3), which recognize viral p25 and viral RNA, respectively [66,67]. TRIM5-p25 heterodimers docked to the pre-integration complex likely suffer degradation by the proteasome thanks to TRIM5 polyubiquitination, thus diminishing the integration of retrotranscribed viral DNA into the host cell genome and, therefore, viral infection [68]. APOBEC, instead, reacts with the nascent viral DNA after retrotranscription, by deaminating cytosines, hence causing detrimental mutations in the viral genome. However, a deaminase-independent mechanism of APOBEC3 restriction has been demonstrated in humans and mice [69]. Both TRIM5 and APOBEC have been considered responsible for the maintenance of the species specific barrier, as well as for the determination of the permissiveness of a given cell type. Although human TRIM5 and APOBEC3 restrict HIV faintly, they restrict distant viruses such as EIAV efficiently. Thus, TRIM5 and APOBEC3 are more potent in restricting heterologous infection than infection caused by the lentivirus of the corresponding particular species [70]. This is of particular interest in infections by SRLV that affect two different species. Lentiviruses have evolved in different ways to evade these restrictive pathways; for instance, the high variability present in particular sites of p25 could be a consequence of the selective pressure exerted by TRIM5 and the binomial Vif-APOBEC3 where the VIF protein present in all the lentiviruses, with the exception of EIAV, interacts with APOBEC molecules, thereby excluding APOBEC from the virion particle and evading restriction mechanisms [71].

So far, ovine and caprine TRIM5 have been described genetically, and some of their molecular species have shown the ability to restrict, *in vitro*, the infection by strain Ev1. In line with studies carried out against HIV using TRIM5 from rhesus monkeys, ovine TRIM5 is able to restrict HIV-2 incoming particles [72]. These encouraging results may open new insights in lentiviral therapy using ovine or caprine molecules of the innate immunity against different lentiviruses, including SRLV.

Studies on small ruminant APOBEC have identified three genes that result in the translation of four proteins, Z1, Z2, Z3 and Z2-Z3, depending on their Zinc domain, responsible for the catalytic activity [73]. SRLV Vif is promiscuous in excluding APOBEC from the virion particles, since it is able to bind APOBEC from different species [74]. Studies are ongoing in our laboratory to gain knowledge on the contribution of APOBEC to SRLV infection. 

Ovine or caprine molecules of the innate immunity may, thus, open new insights in lentiviral therapy and prophylaxis against SRLV. 

## 7. Concluding Remarks

Valuable contributions have been made in the field of SRLV immunization in an attempt to design effective vaccination strategies against viral infections. So far, one of the most promising SRLV immunization studies is that conducted with the naturally attenuated Roccaverano E1 strain, leading to immunomediated protection against heterologous challenge with the pathogenic CAEV Cork strain. Furthermore, the use of SRLV such as *env* and/or *gag* in addition to costimulatory B7 gene-containing plasmids seems to be an attractive approach, at least to reach protection against homologous strains. This strategy could be adapted to pathogenic field strains. Although significant advances have been accomplished in SRLV immunization studies, there are still important questions to solve, such as identifying differences between inflammatory-to-curative and inflammatory-to-clinical responses, before an effective vaccine is considered applicable in field. Future trends involving pseudoviruses and particular innate immunity molecules may open new ways in defense against SRLV infections.
